# Elevated Temperature and Allelopathy Impact Coral Recruitment

**DOI:** 10.1371/journal.pone.0166581

**Published:** 2016-12-07

**Authors:** Raphael Ritson-Williams, Cliff Ross, Valerie J. Paul

**Affiliations:** 1 Smithsonian Marine Station at Fort Pierce, Fort Pierce, FL, United States of America; 2 Department of Biology, University of Hawaii at Manoa, Honolulu, HI, United States of America; 3 Department of Biology, University of North Florida, 1 UNF Drive, Jacksonville, FL, United States of America; Universitat Bremen, GERMANY

## Abstract

As climate change continues to alter seawater temperature and chemistry on a global scale, coral reefs show multiple signs of degradation. One natural process that could facilitate the recovery of reef ecosystems is coral recruitment, which can be influenced by the benthic organisms in a local habitat. We experimentally tested both a global stressor (increased seawater temperature) and a local stressor (exposure to microcolin A, a natural product from a common marine benthic cyanobacterium) to determine how these stressors impacted coral larval sublethal stress, survival and settlement. Larvae of *Porites astreoides* had the same survival and settlement as the controls after exposure to increased temperature alone, but elevated temperature did cause oxidative stress. When exposed to natural concentrations of microcolin A, larval survival and settlement were significantly reduced. When larvae were exposed to these two stressors sequentially there was no interactive effect; but when exposed to both stressors simultaneously, there was a synergistic reduction in larval survival and an increase in oxidative stress more than in either stressor treatment alone. Increased seawater temperatures made larvae more susceptible to a concurrent local stressor disrupting a key process of coral reef recovery and resilience. These results highlight the importance of understanding how interactive stressors of varying spatial scales can impact coral demographics.

## Introduction

As global climate changes the world’s oceans, all marine organisms will face unprecedented abiotic and biotic stressors, especially susceptible are the corals themselves that build tropical reef habitats [1–3]. Recent reviews have focused on how global scale stressors such as elevated seawater temperatures and ocean acidification will impact adult corals [4,5]. Some studies have focused on how multiple stressors might interact to disrupt community dynamics [6,7], and only recently have these studies tested supply side processes such as recruitment, showing relatively few interactive effects of temperature and ocean acidification on coral recruitment [8–10]. Many abiotic and biotic stressors can reduce coral recruitment at any of three critical life-history stages: larval supply, larval settlement and post-settlement survival [11]. Even though inhibitors of coral recruitment such as some algal species have been identified we need to know how these local scale stressors interact with global stressors to disrupt larval ecology.

While much research has studied coral mortality in response to stressors, novel techniques have also been developed to measure sublethal stress. Changing seawater temperature is well studied and is predicted to have huge impacts on coral reefs across the world [1, 2, 4]. Increased seawater temperature is known to induce bleaching and reactive oxygen species (ROS) production in adult corals [12,13], as well as oxidative stress and mortality for some coral larvae [14–17]. Cellular diagnostics have become an important tool to quantify the physiological impacts of sublethal stressors in corals [18–20]. Oxidative stress is commonly measured since it is a ubiquitous stress response that is conserved across numerous taxa [12,21]. Damage to proteins and lipids can be assessed by quantifying levels of protein carbonylation and lipid peroxidation, respectively [22,23]. In addition, the up-regulation of antioxidant enzymatic machinery (e.g., catalase, superoxide dismutase) in response to elevated ROS levels can also indicate sub-lethal stress responses [21]. This fine scale measure of sublethal stress is important in understanding the health of organisms but is rarely utilized in coral larvae studies.

**Fig 1:**
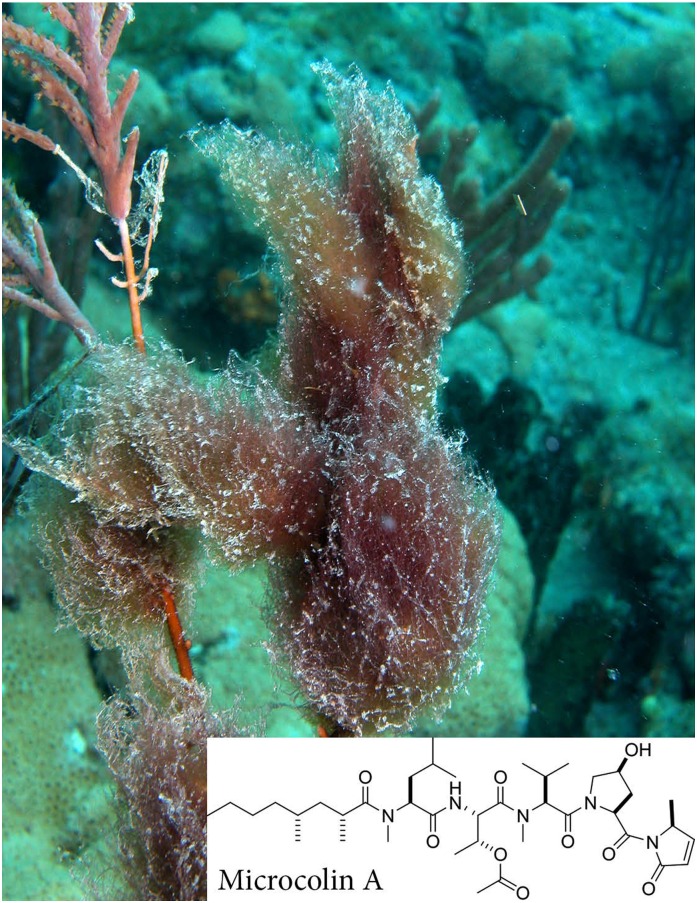
An *in situ* photograph of *Okeania erythroflocculosa* collected from southern Florida reefs, with the structure of microcolin A inset.

Many modern reefs are threatened by increased abundance of macroalgae [24–27], which can stress adult corals [28,29], reduce juvenile coral growth rates [30] and lead to reduced coral fecundity [31]. Live cyanobacteria are also known to inhibit coral larval settlement on settlement substrata [32,33], but the mechanisms of inhibition are not known. Allelopathy (chemically mediated competition among organisms [34]) has been found to drive competition among some sessile marine organisms, including sponge-sponge [35], sponge-coral [36], and macroalgae-coral [29, 37, 38]. All of these studies have incorporated extracts or natural products in an agar or Phytagel strip in comparison to a control strip without added compounds to test allelopathy from isolated secondary metabolites. In the following experiments the compound microcolin A (Fig 1), isolated from *Okeania erythroflocculosa*, previously named *Lyngbya* sp. [39,40], was tested at natural concentrations to determine if its presence on settlement substrata had an allelopathic effect on coral larvae. This method allowed us to test an isolated lipophilic compound without confounding factors such as changes in oxygen concentration due to photosynthesis and respiration [41] release of dissolved organic matter [42], or nutrient cycling [43,44], all of which would be present in experiments with whole cyanobacteria. Since benthic cyanobacteria are prolific producers of secondary metabolites [45,46], we hypothesized that allelopathy is one potential mechanism of cyanobacterial competition with coral larvae. We have chosen to test microcolin A because it is an example of a lipopeptide, which are common in benthic cyanobacteria, it is the major metabolite in *O*. *erythroflocculosa*, and this cyanobacterium can form extensive blooms on southern Florida reefs and is found in the same local habitat as adult *Porites astreoides* [47,48]. Extracts of *O*. *erythroflocculosa* deter feeding by reef fishes [49], likely facilitating cyanobacterial blooms on reefs because grazers avoid consuming benthic cyanobacteria due to their chemical defenses [45–47].

Testing how stressors impact coral population recovery is critical for understanding the processes that shape natural ecosystems in modern habitats. Even though the interactions of multiple stressors can kill individual species, little is known about how multiple stressors interact to disrupt natural ecosystem function [7,50]. This study tested combinations of two types of stressors, a global scale stressor (increased seawater temperature) and a local stressor (microcolin A) independently, sequentially and in combination. We chose to test two potential scenarios since the duration and extent of exposure to elevated seawater temperatures could be quite variable for planktonic larvae: a short term elevated temperature exposure before settlement and a longer temperature exposure concurrent with settlement. After the treatments the coral larvae were assessed for sublethal oxidative stress, survival and settlement. This local to global scale approach to testing processes that drive reef resilience is designed to better understand how different scenarios of future threats will impact modern reefs.

## Results

**Fig 2:**
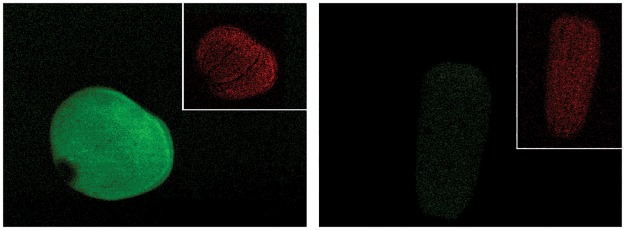
ROS production in larvae of *P*. *astreoides*. ROS production is shown as green fluorescence in the coral larvae. Left panel is a larva that was incubated in seawater at 30.5°C and the right panel is a larva incubated at 27°C, both for 4.5 hours. The larvae are capable of muscular contraction, which enables them to elongate and compress. The images represent two specimens imaged in the same orientation just under different contraction phases, reflecting the plasticity of coral larval morphology. Inset in both panels is the red chlorophyll autofluorescence of the symbiotic zooxanthellae. The two larvae displayed are typical of the sampled groups (n = 15).

When coral larvae of *Porites astreoides* were exposed to +3.5°C seawater temperature for 4.5 hours there was an increased concentration of reactive oxygen species as detected by use of the fluorogenic probe DCFH-DA (Fig 2), directly showing that larvae are exposed to ROS during short term temperature stress. To measure the ecological consequences of temperature stress coupled with a local stressor, two types of temperature stress exposures were tested: one that preceded the local stressor of allelopathy and a temperature stress concurrent with allelopathy.

**Fig 3:**
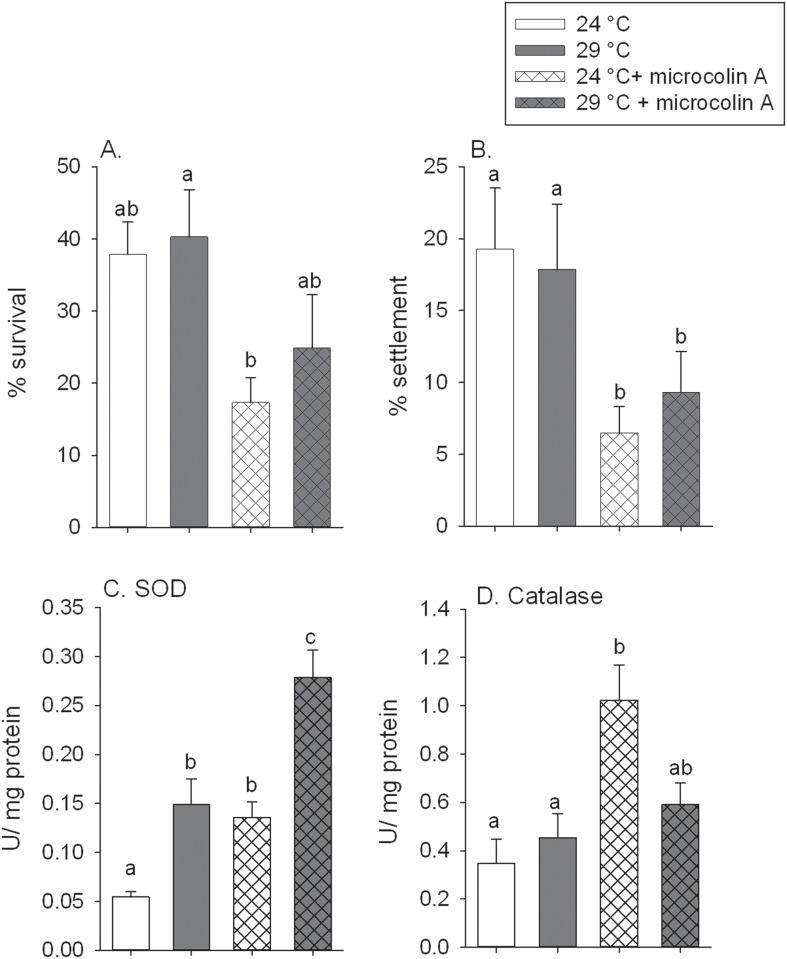
Experiment 1, larval exposure to sequential stressors, 24 hour exposure to elevated seawater temperatures followed by a six day exposure to microcolin A. Bars are untransformed means and error bars are +1 SE, shared letters above the bars indicate means that are not significantly different. (A) The percent of larval survival and (B) the percent of larval settlement after exposure to stress. (C) The activity of superoxide dismutase (SOD) and (D) of catalase (CAT) in the swimming coral larvae at the end of the experiment.

In the first experiment, larvae of *Porites astreoides* were exposed to a short term temperature stress (+5°C) for 24 hours and subsequently given settlement substrata with and without the cyanobacterial compound microcolin A for 6 days. In this first experiment, there was no effect of the temperature treatment on the survival (Fig 3a, p = 0.416) or settlement (Fig 3b, p = 0.789) of larvae. Larvae that were exposed to 29°C seawater compared to 24°C (ambient seawater temperature) had a 2.7 times increase in superoxide dismutase (SOD) activity (Fig 3c, p<0.001) but no change in catalase (CAT) activity (Fig 3d; S1 Table). Larvae of *Porites astreoides* that were then exposed to natural concentrations of microcolin A embedded in agar on settlement tiles after the temperature exposure had reduced survival (Fig 3a, p = 0.003) and settlement (Fig 3b, p = 0.022). The allelopathic compound reduced total survival to less than 25% and total settlement to less than 10% of the larvae supplied in both temperature treatments. After exposure to microcolin A the larvae had a 2.3 times upregulation of the stress enzymes SOD (Fig 3c, p<0.001) and a 2.9 times increase of CAT (Fig 3d, p = 0.001) compared to the controls. There was no significant interaction of the short-term temperature exposure and microcolin A on any of these parameters (S1 Table).

**Fig 4:**
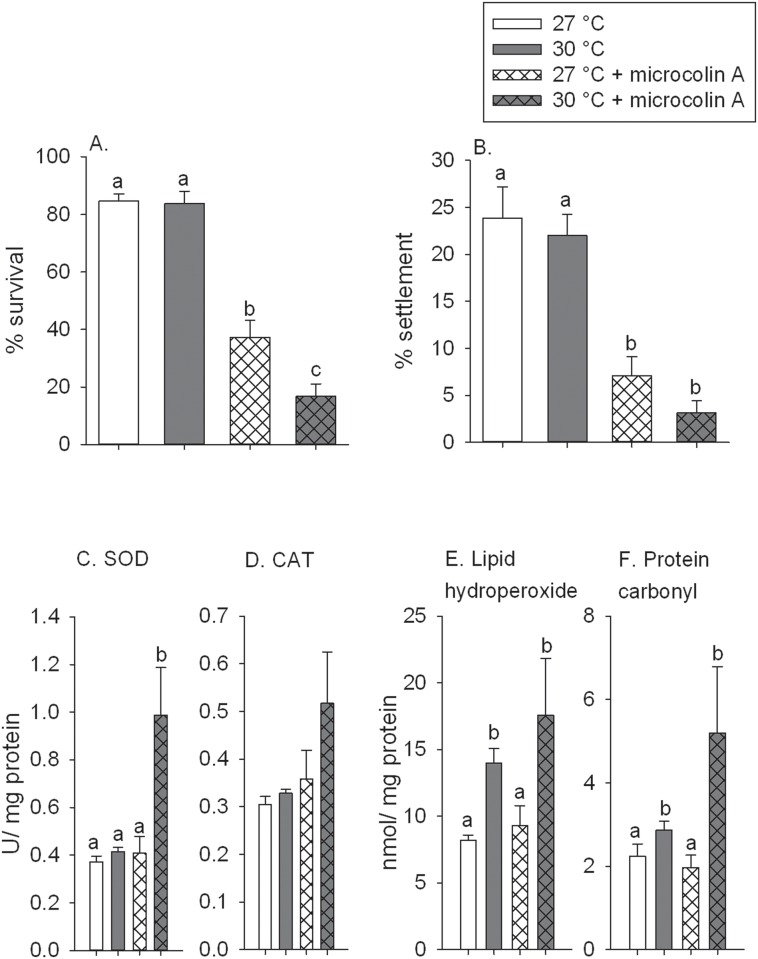
Experiment 2, larval exposure to elevated seawater temperature and microcolin A concurrently. Bars are untransformed means and error bars are +1 SE, shared letters above the bars indicate means that are not significantly different. (A) The percent of larval survival and (B) the percent of larval settlement after a four days exposure to the treatments. (C) The activity of superoxide dismutase (SOD) and (D) catalase (CAT) after four days of exposure. (E) Lipid hydroperoxide and (F) protein carbonylation content following four days of exposure to the treatments.

In the second experiment coral larvae were exposed to + 3°C and microcolin A simultaneously for 4 days. The larvae that were incubated in 30°C seawater, had the same survival and settlement as the control larvae incubated at 27°C (ambient seawater temperature) (Fig 4a and 4b). The temperature treatment alone did not cause increased activities of either SOD or CAT (Fig 4c and 4d). Four days of temperature exposure significantly increased the levels of lipid hydroperoxide by 1.7 times (Fig 4e, p = 0.003) and protein carbonylation content by 1.3 times (Fig 4f, p = 0.007) compared to the controls, indicating oxidative damage to cellular components at higher seawater temperatures. A four-day exposure to agar strips containing microcolin A during settlement significantly reduced larval survival by 43% (Fig 4a, p<0.001) and settlement by 30% compared to the controls (Fig 4b, p<0.001). Microcolin A alone had no effect on the oxidative stress response of these larvae (Fig 4c and 4d).

The four-day exposure to a combination of elevated seawater temperatures and microcolin A caused a significant interaction, with more larval mortality than either stressor alone (Fig 4a, p = 0.037). The combination of stressors reduced settlement to less than 5%, but there was no significant interaction (p = 0.193). Both stressors combined caused a 2.1 times upregulation in the concentration of SOD (Fig 4c, p = 0.023), but had no significant effect on the other biomarkers measured (Fig 4; S2 Table).

## Discussion

Coral recruitment is necessary for reef recovery, but to date there is limited evidence that allelopathy from local benthic organisms can disrupt larval settlement and survival. Isolated compounds have not been previously shown to drive competition in coral larval ecology, but this study shows that microcolin A can reduce survival and inhibit the settlement of larvae of *P*. *astreoides*. Allelopathy with this compound interacted with elevated seawater temperature to reduce local coral recruitment to less than 5% of the larvae supplied (a reduction in settlement of 87% compared to the controls). As climate change continues to impact coral reefs there will be increased frequency of local and global stress events, and understanding interactions with local stressors can provide critical information about fundamental processes such as coral recruitment.

Oxidative stress as a result of thermal stress is well documented in corals [12]. In a preliminary experiment, coral larvae of *Porites astreoides* were exposed to +3.5°C seawater temperature for 4.5 hours, causing increased levels of reactive oxygen species (ROS) when compared to controls, as detected by laser scanning confocal microscopy (Fig 2). Oxidative stress response is a general stress response to multiple types of stressors in adult corals [13], and ROS production can cause extensive damage to cellular components such as membrane lipids [12,21,51]. A short term exposure to elevated seawater temperature caused increased concentration of SOD in the first experiment, and in the second experiment there was increased damage to cellular proteins and lipids, probably due to a relatively long (4 days) exposure to temperature stress. These experiments show different biomarkers were activated through temperature stress, probably due to different durations of thermal exposure. Since the control temperatures were different between the years (due to different ambient seawater temperatures in the Florida Keys), it is difficult to directly compare the oxidative stress of an experimental treatment of +5°C in 2008 to +3°C in 2009. The extent and duration of thermal stress probably has a great influence on the amount of cellular damage in coral larvae. Larvae of *Acropora intermedia* also upregulated SOD in addition to showing elevated levels of lipid hydroperoxide content after exposure to a +6°C increase in seawater temperature for three days [14]. This type of oxidative stress in larvae might cause latent effects that impact coral survival as a juvenile [23]. Multiple studies show that elevated seawater temperatures caused stress in coral larvae, but we are still learning the nuances of how the extent of stress varies with exposure among coral species and how this stress might impact fundamental processes of coral biology.

Other studies have shown that small elevations in seawater temperature (+2–5°C) consistently had no effect on the larval survival and settlement of *P*. *astreoides* [23,52]. Consistently in our temperature treatments there was no effect on larval survival and settlement, but there was a sublethal effect. Both experiments show that elevated temperatures can cause oxidative damage, but corals do have antioxidant mechanisms allowing them to cope with this stressor [12,13]. Prior differences in the concentration of enzymes might also be responsible for the variation in stress response [9], unfortunately the history of these coral colonies or whether maternal effects are influencing stress susceptibility is not known. Dinoflagellates living symbiotically with corals might be an important source of ROS during temperature stress [14,17], and since larvae of *P*. *astreoides* contain these symbionts they may be more susceptible to heat stress than aposymbiotic larvae found in other coral species.

As climate change promises to increase seawater temperatures, many reefs are concurrently experiencing phase shifts from coral to algal dominated communities [24–27]. With increased frequency and cover of macroalgae and cyanobacteria natural processes such as coral recruitment could be impacted. Some studies have found that macroalgae and cyanobacteria can disrupt coral recruitment [32,33,38,52,53,54]. However, the mechanisms of this disruption are multifaceted, and most experiments do not distinguish among different mechanisms of action including space occupation [55], microbial shifts [41,42] and secondary metabolites [38,52,53]. Many studies have focused on marine microbes as indicators of appropriate settlement habitat [56,57,58], but the negative impact of individual microbes on coral settlement is rarely studied. Some natural products and DOM can disrupt natural microbial communities [37,59,60], and it may be that microcolin A can disrupt natural biofilms that would increase settlement. While the experiments in this manuscript do not distinguish this potential mechanism of settlement inhibition, the reduction in larval survival suggests that microcolin A is toxic to coral larvae. Even though a few studies have shown that crude extracts can impact larval recruitment, this is the first study to have tested an isolated secondary metabolite. Previous work with larvae of *P*. *astreoides* showed increased mortality after exposure to crude extracts of brown macroalgae in the genus *Dictyota* incorporated into agar strips and placed on settlement tiles [38]. A 20-hour exposure of *P*. *astreoides* larvae to the brevetoxins in *K*. *brevis* caused decreased larval respiration and increased concentrations of both CAT and lipid hydroperoxides [22]. Water soluble cues from *Padina* sp. reduced the settlement of *Acropora millepora* by 30% [53]. In both of our experiments an isolated compound had a significant effect on larval survival and settlement. We covered approximately 20% of the available settlement substrata with the agar that had microcolin A embedded at natural concentration, which is similar to estimated cyanobacteria coverage during a bloom in Florida [47], and is within the range of cyanobacterial abundance found in an experimental manipulation of turf mats in climate change conditions [61]. Cyanobacteria contain many different types of compounds, but microcolin A is a lipopeptide that typically would not be released into seawater. These agar strips are ecologically relevant by holding microcolin A on the benthos where coral larvae probably contact it during their exploration of the benthos while searching for appropriate settlement habitat. Increased potential for allelopathy due to increasing cyanobacterial abundance on reefs could be an important negative consequence of a phase shift from a coral to algal dominated reef, especially at the vulnerable early life history stages of corals.

Even though there was a strong allelopathic effect of microcolin A on larval survival and settlement, this species of cyanobacteria also contains microcolin B and desacetylmicrocolin B [39], suggesting that our experiments are a conservative estimate of the ecological effect this cyanobacterial species can have on coral larvae. Importantly, our experiments clearly show that allelopathy can kill coral larvae regardless of other features of live cyanobacteria. The magnitude of the effect of microcolin A varied between experiments and this could be due to different exposure durations. In one of our experiments there was an upregulation of SOD in response to microcolin A suggesting this compound also causes oxidative stress. Lipopeptides have been previously shown to induce ROS formation (and concomitant apoptosis) in mammalian cell lines [62,63]. It is probable that similar damage may be occurring in coral larvae when exposed to microcolin A [64]. If blooms of cyanobacteria increase in size and frequency with increased ocean temperatures as predicted [61,65,66], they could greatly increase sublethal stress and larval mortality during coral recruitment.

A combination of stressors has been studied in diverse habitats [6] and is known to be important for coral reefs [7]. But there are relatively few studies that have quantified sublethal stress to better understand the cellular pathways that are impacted by stress. Temperature and ocean acidification combined are known to impact larval corals, and some studies are testing other combinations of stressors. An experiment with *Diploria strigosa* showed that the larvae had decreased survival and settlement in response to increased temperature and the addition of ammonium, but these stressors had an additive not synergistic effect [16]. No synergistic interactions were detected when larvae of *P*. *astreoides* were exposed to temperature and the brown macroalga *Dictyota menstrualis* [52], illustrating that significant interactions of multiple stressors depend on the species tested. Nutrients and temperature impacted the early life history of *Acropora tenuis*, and the type of interaction (additive, synergistic, antagonistic) of these two stressors was dependent on the life history stage of the coral [67]. Synergistic effects have been documented in both marine and terrestrial environments [6,7], and our data provide an example for the potential impact of interactive effects. The corals and larvae in these experiments were maintained at relatively low light levels, however high light is a known stressor for corals and higher levels could also interact with these other stressors to impact recruitment. As marine organisms are increasingly exposed to multiple stressors, a better understanding of sublethal stress and the ensuing susceptibility to other stressors is needed to conserve natural communities.

Coral recruitment is one critical process of reef recovery and resilience that is susceptible to a combination of local and global stressors. Under bloom conditions, cyanobacteria have the potential to create a major demographic bottleneck in coral recruitment through allelopathic effects. This is also true for other species of macroalgae interacting with corals, suggesting that allelopathy is a common driver of competition on reefs [29,38]. The mechanisms of competition between corals and benthic cyanobacteria could also be driven by microbial shifts [42], space occupation [55], a change in local nutrient concentrations due to nutrient cycling [43,44], or shifts in fine scale oxygen concentrations [41], but we show that an isolated secondary metabolite can drive competition regardless of other cyanobacterial features. In addition, sublethal stress caused by elevated seawater temperatures exacerbated the effects of microcolin A on coral larval survival. For increased coral reef resilience it is important that management strategies control local competitive benthic organisms such as macroalgae and cyanobacteria to maximize coral resistance to global threats.

## Materials and Methods

### Collection of *Porites astreoides* larvae

This work was conducted under the permit FKNMS-2008-018 from the Florida Keys National Marine Sanctuary. Each year in May 2007, 2008 and 2009, forty adult colonies of *P*. *astreoides* were collected from the seawall at the base of the Spanish Channel bridge in the lower Florida Keys (GPS N 24° 38.9’ W 81° 19.8’), and transported to Mote’s Tropical Research Laboratory (Summerland Key, FL) where they were maintained in running seawater. Adult colonies were haphazardly collected from a depth of 3–6 meters, and were different colonies each year. To obtain larvae, each colony was placed in an individual 3 L Rubbermaid Grip’s Mix bowl^®^ supplied with continuously running seawater. During the night the larvae spilled over the handles of the bowls into plastic tri-pour beakers with 180 μm mesh bottoms. The water level was held at 15 cm so the larvae remained in the tri-pour beakers until sunrise the next morning when they were counted into the experiments. All larvae were pooled from multiple parents (>25 colonies) to better understand coral recruitment at a population scale and were added to the experimental treatments the same day they were released. Adult colonies were later returned to the collection site and reattached with Z-Spar Splash Zone Compound^®^ underwater epoxy [33].

### Detection of Reactive Oxygen Species (ROS)

A separate set of 50 larvae was collected on May 28, 2007 to examine the effect of thermal stress on the production of reactive oxygen species (ROS). Live larvae were returned to the Smithsonian Marine Station at Fort Pierce and at an age of 2 days and were incubated at 27°C (*n* = 15) or 30.5°C (*n* = 15) for 4.5 hours. Larvae were subsequently placed in 15 ml polystyrene conical tubes containing 10 ml of seawater and 10 μl of dichlorodihydrofluorescein diacetate (DCFH-DA; 5 μM final concentration; Invitrogen, Carlsbad, California, USA) following previous methods [68]. DCFH-DA is a non-fluorescing compound, and when it reacts with cellular esterases the diacetate group is cleaved to yield 2’7’-dichloro-dihydrofluorescein (DCFH). Subsequent oxidation of DCFH by ROS yields the fluorescent product 2’,7’-dichlorofluorescein. Samples were mixed on a rotary shaker in the dark for 15 min, and then washed in filtered seawater to remove any unbound probe. Laser scanning confocal microscopy was employed to visualize the induced production of ROS. A Nikon Eclipse E800 compound microscope (Nikon Instruments, Kanagawa, Japan) outfitted with a Bio-Rad Radiance 2000 laser system (Biorad, Hercules, CA., USA) was used at 20% laser power. Excitation was 488 nm and emission was 525 nm (detection of fluorescein probe) or 580 nm (detection of chlorophyll).

### Experiment 1-Sequential treatment of *Porites astreoides* larvae

Larvae for the first experiment were collected the morning of May 6, 2008 (new moon: May 5). The experiments were set up inside the laboratory in 5 L aquaria that served as water baths. Seven replicate aquaria served as water baths of either ambient temperature (23.5°C ± 0.179 SE) or elevated temperature (29.3°C ± 0.733 SE) with individual ViaAqua^™^ aquarium heaters to increase the seawater bath temperature. Each aquarium contained two 400 ml plastic tri-pour beakers with 220 ml of standing seawater and 200 larvae of *P*. *astreoides*. One replicate (from the treatment with ambient temperature and microcolin A) was lost because all of the larvae had already metamorphosed after the 24 hour temperature incubation. After 24 hours of exposure to the temperature treatments 100 swimming larvae were removed from each replicate and placed in larval containers. Larval containers were made of 800 ml plastic tri-pour beakers that had their bottoms replaced with 180 μm nitex mesh. These larval containers were placed in outdoor flow-through seawater tables and elevated 3 cm off the table to ensure water exchange through the nitex mesh. Each larval container had 4.5 x 4.5 cm terracotta tile that had been pre-conditioned on a patch reef where *P*. *astreoides* was common at a depth of 6 meters for 5 weeks. At the initiation of the experiment a 2 x 3.5 cm agar strip (0.3 cm thick) was attached with a cable tie to the top of the tile covering 50% of the top of the tile (approximately 20% of the potential settlement surface including the top, bottom and sides of tile). All of the larval containers were shaded to reduce ambient light to a maximum daily irradiance of 85 μmol m^-2^s^-1^. This light level was consistent among treatments and controls and was used to ensure the health of the corals and larvae in the shallow seawater tables. Each larval container from an aquarium was randomly assigned either an agar control, or an agar strip that contained microcolin A. The agar strips occupied the same amount of space on the tile, controlling for simple space competition, and only differed in the presence or absence of microcolin A. The control agar strips were composed of 0.625 g agar and 0.625 g carrageenan mixed with 35 ml of distilled water and 5 ml of 95% ethanol. Microcolin A was previously isolated [39], and then dissolved in 5 ml of 95% ethanol and added to the treatment strips at a concentration of 0.03% wet weight (300μg/g wet weight), which is the natural concentration found in the cyanobacterium. Microcolin A does not readily dissolve in seawater and the agar and carrageenan gel was designed to retain the compound as much as possible. We were not testing the diffusion of microcolin A from the agar strip, instead we were testing the presence of allelopathy on the benthos. We confirmed that microcolin A was still present in the agar strips by re-extracting the treatment agar after the experiment and extraction of standing water with agar strips soaking in it for 4 days followed by proton nuclear magnetic resonance (NMR) spectroscopy to detect the presence/absence of microcolin A. Microcolin A was still detected in the agar strips at the end of the experiment and was not detected in the water. After 6 days the larvae were counted for percent survival (swimmers plus settlers) and settlement (larvae that had attached and metamorphosed onto all sides of the tile, the agar strip or the chamber itself). Mortality was calculated by subtracting the total number of larvae that survived from the initial number of larvae because there is no visible remnant of dead coral larvae. All of the swimming larvae were frozen in liquid nitrogen and stored at -80°C for oxidative stress assays. Data were either arc sine square-root or rank transformed to ensure normal distribution and homogenous variances and analyzed with a two-way ANOVA (S1 Table).

### Experiment 2-Concurrent treatment of *Porites astreoides* larvae

Larvae were collected the morning of May 23, 2009 (new moon: May 24). Larval chambers were 10 cm sections of clear acrylic tube (5.5 cm diameter) with 180 μm mesh covering each end. Each larval chamber contained two hundred larvae and a pre-conditioned (at a depth of 6 meters for 6 weeks) 4.5 x 4.5 cm terracotta tile with a 2 x 3.5 cm agar strip attached, as described for experiment 1. Microcolin A was again incorporated at a 0.03% concentration and was still present in the agar strips after the experiment, as determined by proton NMR as described for experiment 1. One larval chamber was placed in one of forty 7 L aquaria that were either the same temperature as the outside flow-through seawater bath (27.15°C ± 0.082 SE) or were heated (30.3°C ± 0.087 SE) using individual ViaAqua^™^ aquarium heaters. All of the control and treatment aquaria were shaded as is described in experiment 1. Each aquarium was randomly assigned a treatment (n = 10). After 4 days the larvae were counted for percent survival and settlement as described above. All of the swimming larvae were frozen in liquid nitrogen and stored at -80°C for oxidative stress assays. Data were either arc sine square-root or rank transformed to assure normal distribution and homogenous variances and analyzed by two-way ANOVA (S2 Table). If there was a significant interaction term each factor was analyzed with a one-way ANOVA [69]. For the response variables with significant interaction terms, the means were confirmed to be different using a Tukey’s post-hoc test.

### Oxidative Stress Assays

In the first experiment, there were enough swimming larvae to conduct oxidative stress assays for catalase and superoxide dismutase on each replicate. In the second experiment, due to low biomass of coral larvae, two random samples from the same treatment were pooled (n = 5). Within one month of each experiment frozen samples of multiple larvae (~ 1 g wet weight) were thawed to room temperature and each extracted in 2.5 ml of buffer (50 mM potassium phosphate buffer (pH 7.0) containing 10% (w/v) polyvinylpolypyrrolidone (PVP)-40, 0.25% Triton X-100). Samples were homogenized with mortar and pestle and centrifuged at 16,000 x g for 15 minutes. The resulting supernatants were quantified for protein content and assayed for CAT and SOD. Total soluble protein (TSP) was quantified with the Quick Start^™^ Bradford Protein Assay Kit (Bio-Rad, Hercules, California, USA). CAT activity was assayed with a BIOXYTECH^®^ Catalase-520^™^ kit (Oxis International Inc., Foster City, California, USA), and SOD activity was monitored with a BIOXYTECH^®^ SOD-525^™^ kit (Oxis International Inc.) according to the manufacturers’ instructions. Enzyme activity was normalized to protein content and units were expressed as U mg protein^-1^. The samples from the second experiment were also analyzed for the presence of protein carbonylation and lipid peroxidation using a Protein Carbonyl Assay Kit and Lipid Hydroperoxide Assay Kit, respectively (Cayman Chemical, Anne Arbor, MI, USA). Data were either arc sine square-root or rank transformed to assure normal distribution and homogenous variances and were analyzed with a two-way ANOVA (S1 and S2 Tables).

## Supporting Information

S1 Data The raw data generated during the experiments and subsequent analysis. (XLS)

S1 Table Statistical analysis of Experiment 1, a sequential treatment of larvae of *Porites astreoides*. (DOC)

S2 Table Statistical analysis of Experiment 2, a simultaneous treatment of larvae of *Porites astreoides*. Any terms with a significant interaction were analyzed within each of the factors with a one-way ANOVA, shown at the bottom of the table. N.S. indicates p<0.05 factors that were not significant after further analysis.
(DOCX)
