# Supplementary material for: Elevated Temperature and Allelopathy Impact Coral Recruitment
Source: PLoS One. 2016 Dec 7;11(12):e0166581. doi: 10.1371/journal.pone.0166581 (PMC5142781; doi:10.1371/journal.pone.0166581)
Supplement: S1 Table — (DOC) [file pone.0166581.s002.doc]

Supplementary Table 1. Statistical analysis of Experiment 1, a sequential treatment of larvae of *Porites astreoides*.

Two-Way Analysis of Variance

**% survival**-arcsin square-root transformed DF Mean-Square F ratio p-value

| Microcolin A | 1 | 0.311 | 10.67 | 0.003 |
| --- | --- | --- | --- | --- |
| Temperature | 1 | 0.020 | 0.69 | 0.416 |
| Interaction | 1 | 0.006 | 0.20 | 0.657 |
| Error | 23 | 0.029 |  |  |

**% settlement**-arcsin square-root transformed

| Microcolin A | 1 | 0.160 | 6.00 | 0.022 |
| --- | --- | --- | --- | --- |
| Temperature | 1 | 0.002 | 0.07 | 0.789 |
| Interaction | 1 | 0.004 | 0.16 | 0.688 |
| Error | 23 | 0.027 |  |  |

**Superoxide Dismutase**-log (x) transformed

| Microcolin A | 1 | 0.861 | 23.44 | <0.001 |
| --- | --- | --- | --- | --- |
| Temperature | 1 | 0.868 | 23.63 | <0.001 |
| Interaction | 1 | 0.007 | 0.19 | 0.670 |
| Error | 23 | 0.037 |  |  |

**Catalase**-log (x) transformed

| Microcolin A | 1 | 0.907 | 13.51 | 0.001 |
| --- | --- | --- | --- | --- |
| Temperature | 1 | 0.022 | 0.33 | 0.573 |
| Interaction | 1 | 0.252 | 3.75 | 0.065 |
| Error | 23 | 0.067 |  |  |
