# Supplementary material for: Elevated Temperature and Allelopathy Impact Coral Recruitment
Source: PLoS One. 2016 Dec 7;11(12):e0166581. doi: 10.1371/journal.pone.0166581 (PMC5142781; doi:10.1371/journal.pone.0166581)
Supplement: S2 Table — Any terms with a significant interaction were analyzed within each of the factors with a one-way ANOVA, shown at the bottom of the table. N.S. indicates p<0.05 factors that were not significant after further analysis. (DOCX) [file pone.0166581.s003.docx]

Supplementary Table 2. Statistical analysis of Experiment 2, a simultaneous treatment of larvae of *Porites astreoides*. Any terms with a significant interaction were analyzed within each of the factors with a one-way ANOVA, shown at the bottom of the table. N.S. indicates p<0.05 factors that were not significant after further analysis.

Two-Way Analysis of Variance

**% survival**-arcsin square-root transformed DF Mean-Square F ratio p-value

| Microcolin A | 1 | 4.527 | 126.58 | <0.001 |
| --- | --- | --- | --- | --- |
| Temperature | 1 | 0.149 | 4.15 | 0.049 N.S. |
| Interaction | 1 | 0.169 | 4.72 | 0.037 |
| Error | 36 | 0.036 |  |  |

**% settlement**-arcsin square-root transformed

| Microcolin A | 1 | 0.900 | 69.48 | <0.001 |
| --- | --- | --- | --- | --- |
| Temperature | 1 | 0.039 | 3.01 | 0.091 |
| Interaction | 1 | 0.023 | 1.76 | 0.193 |
| Error | 36 | 0.013 |  |  |

**Superoxide Dismutase**-rank transformed

| Microcolin A | 1 | 0.466 | 8.20 | 0.011 N.S. |
| --- | --- | --- | --- | --- |
| Temperature | 1 | 0.485 | 8.52 | 0.010 N.S. |
| Interaction | 1 | 0.361 | 6.35 | 0.023 |
| Error | 16 | 0.057 |  |  |

**Catalase**-rank transformed

| Microcolin A | 1 | 0.073 | 3.78 | 0.070 |
| --- | --- | --- | --- | --- |
| Temperature | 1 | 0.042 | 2.14 | 0.163 |
| Interaction | 1 | 0.023 | 1.18 | 0.293 |
| Error | 16 | 0.019 |  |  |

**Lipid hydroperoxidase**-log (x) transformed

| Microcolin A | 1 | 0.009 | 0.04 | 0.537 |
| --- | --- | --- | --- | --- |
| Temperature | 1 | 0.278 | 12.12 | 0.003 |
| Interaction | 1 | <0.001 | 0.01 | 0.926 |
| Error | 16 | 0.023 |  |  |

**Protein Carbonyl**-log (x) transformed

| Microcolin A | 1 | 0.020 | 0.66 | 0.427 |
| --- | --- | --- | --- | --- |
| Temperature | 1 | 0.294 | 9.58 | 0.007 |
| Interaction | 1 | 0.079 | 2.58 | 0.128 |
| Error | 16 | 0.031 |  |  |

One-Way Analysis of Variance- for the factors with significant interaction terms

**% survival**-arcsin square-root transformed DF Mean-Square F ratio p-value

| +/- Microcolin A at 27 °C | 1 | 1.474 | 47.37 | <0.001 |
| --- | --- | --- | --- | --- |
| +/- Microcolin A at 30 °C | 1 | 3.222 | 79.72 | <0.001 |
| 27 °C vs. 30 °C without microcolin A | 1 | <0.001 | 0.01 | 0.916 |
| 27 °C vs. 30 °C with microcolin A | 1 | 0.317 | 7.25 | 0.015 |

**Superoxide Dismutase**- not transformed except for 27 °C vs. 30 °C with microcolin A (rank transform)

| +/- Microcolin A at 27° C | 1 | 0.003 | 0.23 | 0.641 |
| --- | --- | --- | --- | --- |
| +/- Microcolin A at 30° C | 1 | 0.297 | 25.0 | 0.001 |
| 27 °C vs. 30 °C without microcolin A | 1 | 0.005 | 1.85 | 0.211 |
| 27 °C vs. 30 °C with microcolin A | 1 | 0.842 | 7.56 | 0.025 |
